# Prospective clinical study to evaluate the survival of two-piece zirconia implants: a single-center study. 36-month results

**DOI:** 10.1186/s40729-026-00707-0

**Published:** 2026-08-19

**Authors:** Marie-Elise Jennes, Zhen Mao, Ece Atay, Insa Herklotz, Margarita Bessonova, Jeremias Hey, Florian Beuer

**Affiliations:** 1 Department of Prosthodontics, Geriatric Dentistry and Craniomandibular Disorders, Charité – University Medical Center Berlin, corporate member of Freie Universität Berlin and Humboldt-Universität zu Berlin, Assmanshauser Straße 4-6, 14197 Berlin, Germany; 2 Private Dental Practice, Amalienpark 1, 13187 Berlin, Germany; 3 Private Dental Practice, Ährenweg 1, 32469 Petershagen, Germany; 4 Department of Prosthetic Dentistry, University School of Dental Medicine, Martin Luther University Halle-Wittenberg, Magdeburger Str. 16, 06112 Halle, Germany

**Keywords:** Ceramic implants, Zirconia, Two-piece implants, Implant fracture, Screw-retained restoration, Dental implants, Implant survival, PEKK abutment

## Abstract

**Purpose:**

To evaluate the 36-month survival and clinical outcomes of two-piece zirconia implants restored with screw-retained glass-ceramic crowns on PEKK abutments.

**Methods:**

This prospective single-center clinical study evaluated implant survival as the primary outcome, with secondary outcomes including peri-implant soft tissue parameters, marginal bone level changes, and patient-reported outcome measures. Twenty-four patients received single CERALOG Hexalobe two-piece zirconia implants manufactured by ceramic injection molding (CIM) in healed sites. After 6-month submerged healing, implants were restored with provisional screw-retained crowns on PEKK abutments, followed by definitive lithium disilicate crowns (IPS e.max Press) on PEKK abutments secured with titanium abutment screws (25 Ncm). Clinical and radiographic examinations were performed at implant placement, re-entry, definitive loading, and at 12-, 24-, and 36-month follow-ups. Implant survival was analyzed using Kaplan–Meier estimates.

**Results:**

Of 23 evaluable implants, nine failed within the first 12 months: six due to failed osseointegration (two at re-entry, three during provisional restoration, one after definitive loading) and three due to implant fracture (one during provisional restoration, two after definitive loading). Kaplan-Meier survival rate was 60.9% (95% CI; 41–81%) at 12 months. No additional failures occurred between 12 and 36 months. Peri-implant soft tissue parameters of surviving implants showed favorable trends (PPD: 2.7 ± 0.7 mm at 12 months, 2.0 ± 0.5 mm at 24 months, 1.9 ± 0.8 mm at 36 months; BOP: 26%, 21%, and 18%, respectively; MGI: 0.38 ± 0.36 at 12 months, 0.40 ± 0.76 at 24 months, 0.21 ± 0.48 at 36 months). At the implant level, peri-implant mucositis was diagnosed in 7 of 13 implants (53.8%) at 36 months; no peri-implantitis was observed throughout the observation period. Mean DIB was 1.9 ± 0.6 mm at definitive loading, 1.6 ± 0.5 mm at 24 months, and 1.5 ± 0.9 mm at 36 months. One abutment screw loosening at 24 months was successfully managed. Patients with surviving implants reported high satisfaction scores (overall satisfaction: 5.0 ± 0.0 at 36 months).

**Conclusions:**

The 39.1% early failure rate within the first 12 months was followed by stable marginal bone levels through 36 months in implants that achieved successful osseointegration. However, peri-implant mucositis remained prevalent at 36 months, underlining the need for peri-implant maintenance. The early post-loading period represents the critical determinant of long-term implant survival in this system. Extended follow-up through 60 months is ongoing.

## Background

Two-piece zirconia implants have emerged as an alternative to titanium implants, offering potential advantages in patients with thin mucosal phenotypes or high smile lines [1]. A recent systematic review reported a pooled survival rate of 96.3% for two-piece zirconia implants, although follow-up periods ranged considerably [2] and medium- to long-term evidence remains limited [3]. In the previously published 12-month follow-up of this cohort, CERALOG Hexalobe two-piece zirconia implants manufactured by ceramic injection molding (CIM) (ALTATEC GmbH, Wimsheim, Germany) and restored with screw-retained lithium disilicate crowns on PEKK abutments showed a 12-month survival rate of 60.9% [4]. Most failures were related to osseointegration problems (67%), while the remaining losses resulted from implant fractures (33%). Notably, all implant losses occurred prior to or shortly after definitive loading [4]. These observations raise a clinically important question: if early failures in two-piece zirconia implants are predominantly related to osseointegration problems, do implants that survive this critical period demonstrate stable medium-term outcomes?

Large-scale epidemiological data demonstrate that approximately 70% of all dental implant failures occur within the first year after placement, with the rate of implant loss decreasing substantially thereafter [5]. Importantly, this pattern is not limited to titanium implants. A PRISMA-based systematic review on zirconia implants restored with fixed prostheses reported most implant losses occurred within the first year, especially during healing, and that survival curves were largely stable thereafter [6].

Early implant failures are generally defined as losses occurring before or shortly after prosthetic loading [7]. From a biological perspective, early failure results from an inability to establish intimate bone-to-implant contact, meaning that bone healing after implant insertion is impaired. The mechanisms that normally lead to wound healing by means of bone apposition fail, and instead an epithelial downgrowth leads to the formation of fibrous scar tissue around the implant, which can result in implant mobility or loss [8]. In contrast, late failures occur after successful osseointegration and functional loading, with peri-implantitis representing the predominant cause [9].

The present study extends these observations to 36 months post-loading, with the primary objective of determining whether additional implant failures occurred beyond the initial 12-month period [4]. Secondary objectives included longitudinal assessment of peri-implant tissue parameters, marginal bone levels, and patient-reported outcome measures in surviving implants.

## Methods

This article reports the 36-month follow-up results of patients treated with CERALOG Hexalobe two-piece, CIM-manufactured zirconia implants (ALTATEC GmbH, Wimsheim, Germany). The study adhered to the principles outlined in the Declaration of Helsinki for experiments involving human subjects [10] and followed the STROBE guidelines for observational studies [11]. The study protocol was registered with the German Clinical Trials Register (Deutsches Register Klinischer Studien, DRKS; registration number DRKS00012469). All treatments and investigations were performed at the Department of Prosthodontics, Geriatric Dentistry and Craniomandibular Disorders ofCharité – University Medical Center Berlin, Germany.

The complete study design, including detailed eligibility criteria, surgical protocols, prosthetic workflows, and measurement procedures, has been previously published [4]. In brief, definitive restorations consisted of screw-retained lithium disilicate crowns (IPS e.max Press, Ivoclar, Schaan, Liechtenstein) on PEKK abutments (ALTATEC GmbH, Wimsheim, Germany), secured with titanium abutment screws tightened to 25 Ncm according to the manufacturer’s recommendation. All implants and components were CE-marked and used within the indications recommended by the manufacturer.

### Follow-up protocol

The primary objective was to assess implant survival across the entire observation period from implantation to 36 months, with follow-up visits scheduled at definitive loading, 12 months, 24 months, 36 months, and annually thereafter through 60 months.

Implant survival was defined as the implant remaining in situ throughout the observation period without removal, irrespective of biological or technical complications. No formal success rate was calculated; instead, peri-implant tissue conditions and complications were reported descriptively for the surviving implant cohort.

Secondary outcomes included longitudinal assessment of:

- Distance from implant shoulder to first bone contact (DIB).
- Soft tissue parameters (probing pocket depth (PPD), bleeding on probing (BOP), modified gingival index (MGI)).
- Patient-reported outcome measures (PROMs).
- Nature and frequency of all adverse events.

Clinical and radiographic examinations were conducted at 24- and 36-month follow-ups. Standardized periapical radiographs were obtained using individualized bite blocks and film-holder positioning templates to ensure reproducible projection geometry across visits. The distance from the implant shoulder to the first bone-to-implant contact (DIB) was measured at the mesial and distal aspects of each implant. To correct for radiographic distortion, the known intraosseous implant length (8–10 mm) was used as an internal reference, and the pixel-to-millimeter conversion factor was determined individually for each radiograph based on the radiographically measured implant length. Detailed assessment protocols are described in the initial publication [4].

### Statistical evaluation

Descriptive statistics were performed using IBM SPSS Statistics (Version 29.0, IBM, Armonk, NY, USA). Implant survival was analyzed for all 23 evaluable implants across the entire observation period from implantation to 36 months using Kaplan-Meier survival analysis. Mean values and standard deviations were calculated for all continuous variables.

## Results

### Patient cohort and implant characteristics

Between April 2017 and March 2019, 24 patients (11 female, 13 male; mean age 48.4 years, range 27–77 years) received single two-piece zirconia implants, as previously described [4]. The implants replaced 13 premolars and 11 molars, with 13 placed in the maxilla and 11 in the mandible. All implants (diameter 4.0/4.5 mm; intraosseous lengths 8 mm [*n* = 8] or 10 mm [*n* = 16]) achieved primary stability at placement. One patient (#24) was lost to follow-up immediately after implantation without attending any subsequent examinations, resulting in 23 evaluable implants for survival analysis.

### Implant survival and temporal distribution of failures

Of the 23 evaluable implants, nine failures occurred exclusively within the first 12 months post-loading. Two implants (#13, #19) failed to achieve osseointegration and were identified at the re-entry surgery. Four implants (#6, #8, #9, #16) were lost due to mobility: three during the provisional restoration phase and one four months after definitive crown placement. Three implants (#3, #17, #20) fractured in the coronal third: one during provisional restoration and two within three months following definitive loading. In all cases of mobility-related failure, aseptic connective tissue encapsulation was observed, indicating incomplete osseointegration. Radiographic examination revealed circumferential radiolucency around all mobile implants. All failed implants were subsequently replaced with titanium implants (ALTATEC GmbH, Wimsheim, Germany) following a 3-month healing period and restored with screw-retained crowns. Detailed information about the lost implants can be seen in Table 1.

At 24 months, 12 patients attended the clinical examination. Two patients were temporarily lost to follow-up but confirmed by telephone that their implants remained functional and free of complications. At 36 months, 13 patients attended the clinical examination. One additional patient who had relocated confirmed implant survival and the absence of adverse events by telephone.

**Fig. 1:**
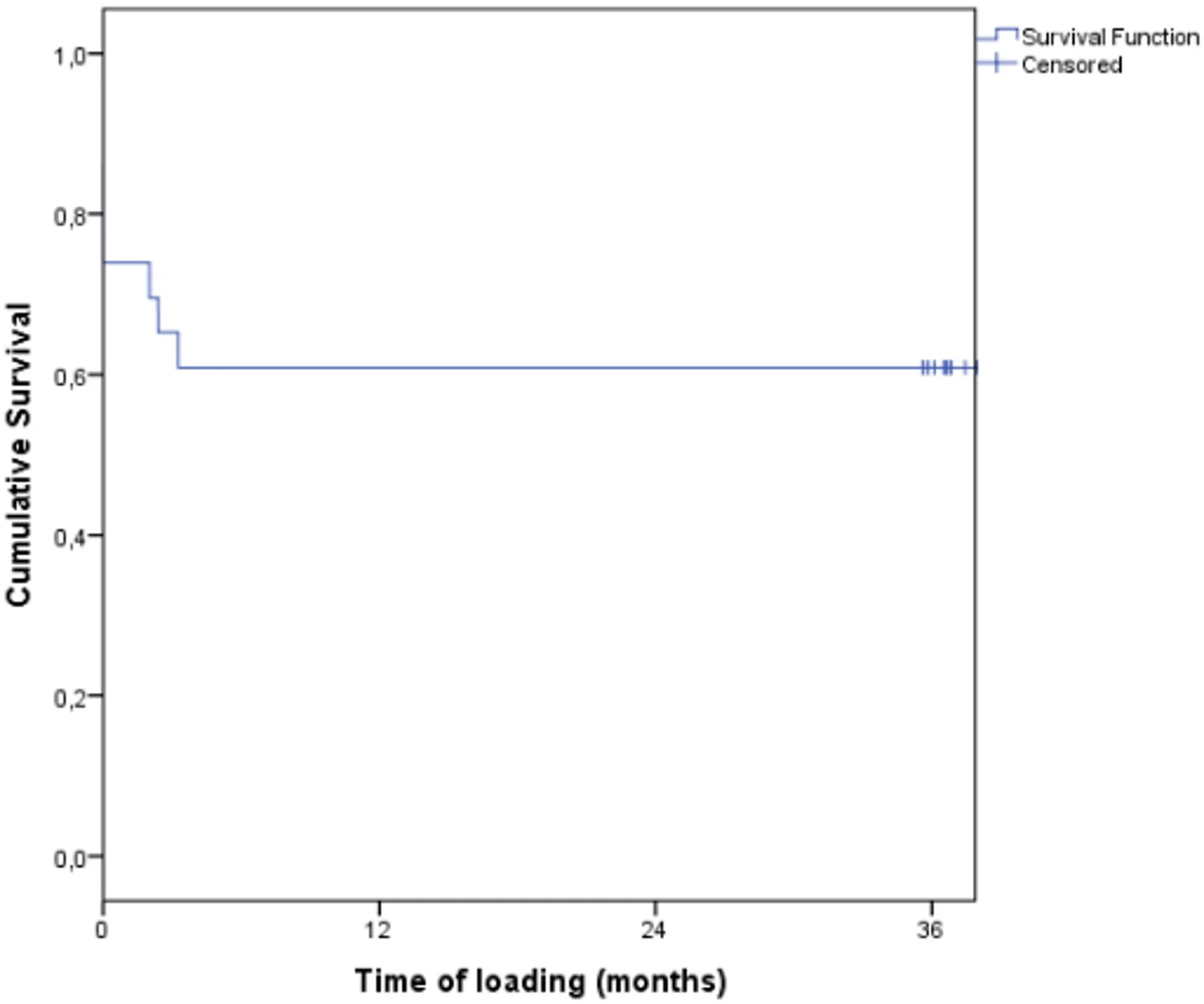
Cumulative implant survival of the 23 evaluable implants in relation to definitive loading. The x-axis is aligned to the time of definitive loading to illustrate the temporal clustering of failures. Six implants failed before definitive loading, resulting in a cumulative survival of 73.9% at loading. Three additional failures occurred within the first four months after definitive loading, reducing cumulative survival to 60.9% (95% CI; 41–81%), which remained unchanged through 36 months

Kaplan-Meier analysis of all 23 evaluable implants demonstrated a cumulative survival probability of 60.9% at 12 months post-loading (95% CI; 41–81%). At the time of definitive loading, 17 of 23 implants remained in situ, corresponding to a cumulative survival of 73.9%; three additional failures occurred within the first four months after definitive loading. No further implant losses were observed between 12 and 36 months post-loading (Fig. 1).

**Table 1:** Detailed characteristics of failed implants

Patient no.	Region	Gender	Smoking status	Bone quality	Failure pattern	Time of loss
#3	36	f	Never	D2	Implant fracture	During provisional restoration
#6	26	f	Never	D2	Failed osseointegration	After definitive crown placement
#8	26	f	Never	D3	Failed osseointegration	During provisional restoration
#9	24	m	Never	D2	Failed osseointegration	During provisional restoration
#13	46	m	Never	D2	Failed osseointegration	At re-entry
#16	34	m	Former	D2	Failed osseointegration	During provisional restoration
#17	25	m	Never	D2	Implant fracture	After definitive crown placement
#19	14	m	Current < 10/day	D2	Failed osseointegration	At re-entry
#20	46	f	Never	D1	Implant fracture	After definitive crown placement

Only one failed implant occurred in a current smoker (patient #19). Failures presenting with implant mobility were interpreted as incomplete osseointegration; all were associated with aseptic connective tissue encapsulation and circumferential radiolucency. All failures occurred before or within the first four months after definitive loading

### Soft tissue parameters

Soft tissue parameters demonstrated overall stability and favorable trends throughout the observation period (Table 2). Mean PPD showed progressive improvement, decreasing from 2.7 ± 0.7 mm at 12 months to 2.0 ± 0.5 mm at 24 months and 1.9 ± 0.8 mm at 36 months. 

At 24 months, one site (1.4%) presented with a PPD of 4 mm, and no site showed a PPD of 5 mm or more. At 36 months, three sites (3.8%) presented with a PPD of 4 mm, and one site (1.3%) showed a PPD of 5 mm or more. Bleeding on probing initially decreased from 26% at 12 months to 21% at 24 months and 18% at 36 months, approaching baseline levels with 17.7% at definitive loading [4]. When evaluated at the implant level, peri-implant mucositis was defined as the presence of bleeding on gentle probing without progressive bone loss [12]. An implant was diagnosed with mucositis if bleeding on probing was recorded at one or more of the six probing sites. Using this criterion, peri-implant mucositis was diagnosed in 7 of 13 clinically examined implants (53.8%) at 36 months, decreasing from 11 of 16 implants (68.8%) at the time of definitive loading. No peri-implantitis was diagnosed throughout the observation period.

The modified gingival index remained consistently low, with mean values of 0.34 ± 0.46 at definitive loading, 0.38 ± 0.36 at 12 months [4], 0.40 ± 0.76 at 24 months, and 0.21 ± 0.48 at 36 months.

**Table 2:** Soft tissue parameters of surviving implants

Parameter	24-month (*n* = 12 implants)	36-month (*n* = 13 implants)
PPD (mm), mean ± SD	2.0 ± 0.5	1.9 ± 0.8
PPD = 4 mm, n (%)	1 (1.4%)	3 (3.8%)
PPD ≥ 5 mm, n (%)	0 (0%)	1 (1.3%)
BOP (%)	21%	18%
MGI, mean ± SD	0.40 ± 0.76	0.21 ± 0.48

PPD, Probing pocket depth; BOP, Bleeding on probing; MGI, Modified gingival index

### Radiographic assessment of marginal bone levels

The mean distance from the implant shoulder to first bone contact (DIB) at definitive loading was 1.9 ± 0.6 mm. At the 12-month follow-up, this decreased to 1.4 ± 0.6 mm, indicating bone apposition of approximately 0.5 mm as previously reported [4]. At the 24- and 36-month follow-ups, mean DIB values were 1.6 ± 0.5 mm and 1.5 ± 0.9 mm, respectively, indicating stable marginal bone levels after the initial remodeling phase. The 36-month DIB analysis was based on 12 implants, as no standardized periapical radiograph was available for one patient at this time point. With the exception of one implant (patient #5), no progressive bone loss was observed in any surviving implant.

### Oral hygiene maintenance

Patient oral hygiene was documented descriptively at each follow-up visit using a study-specific four-category clinical rating based on the overall clinical impression of oral hygiene (1 = excellent, 2 = good, 3 = fair, 4 = poor) (Table 3). At 24 months, 10 of 12 examined patients demonstrated excellent or good oral hygiene, whereas 2 patients (#14 and #18) were rated as having poor oral hygiene. In patient #18, poor oral hygiene was associated with increased peri-implant mucosal inflammation and limited oral hygiene capability due to newly developed dementia; however, no progressive radiographic bone loss was observed. Patient #14 showed no clinical or radiographic abnormalities at the implant site despite the poor oral hygiene rating. At 36 months, 10 of 13 patients maintained excellent or good oral hygiene, with 1 patient (#5) showing fair and 2 patients (#18 and #23) showing poor oral hygiene. In patients #18 and #23 persistent peri-implant mucositis was observed without radiographic evidence of progressive bone loss. Across the entire observation period only patient #5 exhibited an increase in DIB consistent with marginal bone loss. All nine early implant failures occurred in patients with excellent or good oral hygiene at baseline and prior to implant loss.

**Table 3:** Oral hygiene index of examined patients at different visits

Oral hygiene index	24-month	36-month
1 (excellent)	3	5
2 (good)	7	5
3 (fair)	0	1
4 (poor)	2	2
Total	12	13

Oral Hygiene Index: 1 = Excellent, 2 = Good, 3 = Fair, 4 = Poor

Two patients (#2, #5) did not attend the 24-month recall. One patient (#7) did not attend the 36-month recall.

### Technical and biological complications

One technical complication occurred during the observation period. Patient #18 presented with abutment screw loosening at the 24-month examination. The titanium abutment screw was replaced and re-tightened according to manufacturer specifications. No further complications were observed with this implant through the 36-month follow-up, and peri-implant tissues remained healthy.

No additional technical complications (ceramic fracture, chipping, or abutment fracture) were documented during the entire 36-month observation period. Similarly, no peri-implantitis was diagnosed, and apart from the marginal bone loss observed in patient #5, no further biological complications occurred beyond the early failures described above.

### Patient- reported outcome measures

PROM questionnaires for patients with surviving implants at 24 months (*n* = 12) demonstrated high satisfaction scores on a 5-point Likert scale: comfort, chewing function, taste sensation, fit, aesthetic appearance, and overall satisfaction. At 36 months (*n* = 13), satisfaction scores remained stable or improved (Table 4).

**Table 4:** Patient-reported outcome measures (PROMs) at 24- and 36-month follow-up

	24-month (*n* = 12) Mean ± SD	36-month (*n* = 13) Mean ± SD
Comfort	4.75 ± 0.45	4.92 ± 0.29
Chewing function	4.92 ± 0.29	4.92 ± 0.29
Taste sensation	4.92 ± 0.29	4.92 ± 0.29
Fit	5.00 ± 0.00	4.92 ± 0.29
Aesthetic appearance	4.83 ± 0.39	4.92 ± 0.29
Overall satisfaction	4.83 ± 0.39	5.00 ± 0.00

Values are presented as mean ± standard deviation

Likert scale: 1 = Very dissatisfied, 2 = Dissatisfied, 3 = Neutral, 4 = Satisfied, 5 = Very satisfied

Two patients (#2, #5) did not attend the 24-month recall. One patient (#7) did not attend the 36-month recall.

## Discussion

This prospective study extends the previous 12-month findings [4] to a 36-month follow-up, revealing a clinically significant temporal pattern in implant failure. All nine implant failures occurred exclusively before or within the first four months after definitive loading. No additional losses were recorded between 12 and 36 months, and the cumulative survival rate remained stable at 60.9% (14/23 implants) throughout the entire observation period. This finding suggests that once successful osseointegration is established, two-piece zirconia implants with screw-retained restorations on PEKK abutments can demonstrate stable marginal bone levels over the medium term, although peri-implant mucositis remained prevalent at 36 months. Since the previous publication of the 12-month results, the clinical evidence base for the two-piece zirconia CIM-manufactured CERALOG Hexalobe implant system has expanded, yet remains limited. To date, only five clinical in-vivo studies, including the present investigation, have evaluated this implant system [4, 13–16], with a combined total of only 141 implants evaluated across all five studies. Sterzenbach et al. [13] conducted a randomized controlled trial comparing 30 CERALOG Hexalobe implants with 30 titanium implants and reported a cumulative zirconia implant survival rate of 89.3% after three years. Six of 30 zirconia implants were lost due to incomplete osseointegration. Three failed before prosthetic loading and were successfully revised; the remaining three were lost after crown placement (two within the first year, one after 30 months). All titanium implants survived. Since the three revised implants were excluded from the survival analysis, the reported cumulative survival rate was 89.3%; including all six losses, the rate would be 80.0% (24/30). Trimpou et al. [15] investigated a modified version of the system (CERALOG Plus Hexalobe). These CIM-manufactured implants featured a nano-structured surface coating (Ra 1.6 μm) and were restored according to an immediate restoration protocol. The authors reported a 12-month survival rate of 80.0%. Of 26 implants placed in 23 patients, five could not be immediately restored due to insufficient insertion torque (< 30 Ncm) and were allocated to submerged healing; four implants were lost due to loosening at 6, 9, 11, and 20 weeks after immediate restoration, while all five submerged implants survived. As all failures occurred exclusively in the immediately restored group, the authors concluded that the immediate restoration protocol itself may have represented an additional risk factor for early implant loss. Importantly, however, Trimpou et al. [15] did not investigate the original CERALOG Hexalobe surface, but a surface-modified derivative of the system. Accordingly, these findings cannot be considered a direct clinical comparison with the original CERALOG Hexalobe implants evaluated in the present study. Giulini et al. [16] evaluated 32 CERALOG implants in 17 patients in a prospective study and reported 100% implant survival after a mean follow-up of two years. Sala et al. [14] evaluated a mixed cohort of one-piece and two-piece CERALOG implants (29 implants in 18 patients) and reported a five-year survival rate of 73% at the implant level for the two-piece subgroup, with all losses occurring during the osseointegration phase. In both publications, the implants were described as CERALOG implants, but the precise commercial subdesignation was not specified in a way that allows unambiguous assignment to the original CERALOG Hexalobe nomenclature.

Furthermore, the prosthetic protocols varied across the five studies. The present study and Sterzenbach et al. [13] restored implants with screw-retained lithium disilicate crowns (IPS e.max) on PEKK abutments. Trimpou et al. [15] used screw-retained monolithic zirconia crowns (Katana Zirconia, Kuraray Noritake) on PEKK abutments. In contrast, Giulini et al. [16] cemented monolithic all-ceramic crowns on PEKK abutments with a semi-permanent cement. Sala et al. [14] initially followed the manufacturer’s recommendation to use polymer crowns but switched to porcelain zirconia crowns after observing crown fractures with the original material. Across all five studies, osseointegration failure represents the predominant cause of implant loss, and no implant fractures were reported in any study other than ours.

As discussed by Trimpou et al. [15], these osseointegration failures may be related to the surface characteristics inherent to the CIM manufacturing process. Beger et al. [17] demonstrated that the CERALOG implant surface, produced via CIM, exhibits a uniformly distributed, droplet-like microstructure without subsequent surface modification. A recent comparative analysis of commercially available zirconia implant surfaces by Jin et al. [18] highlighted that most competing systems employ dedicated surface treatments such as sandblasting, acid-etching, or ultraviolet photofunctionalization to enhance the biological response, whereas the CIM surface does not undergo any subsequent surface modification. While the measured roughness values (Sa ~ 1.22 μm) fall within the moderately rough range considered favorable for osseointegration, the absence of post-processing surface treatment may result in a less ideal microtopography for initial bone cell attachment.

Three implants in our cohort fractured in the coronal third after loading, a failure mode not reported in any other CERALOG clinical study [13–16]. A critical distinction is the abutment screw material: this study exclusively used titanium screws (25 Ncm), whereas Sterzenbach et al. [13] and Sala et al. [14] employed gold screws (15 Ncm), both in accordance with the respective manufacturer’s recommendations. Giulini et al. [16] also used gold alloy screws but applied a torque of 25 Ncm, exceeding the manufacturer’s recommendation of 15 Ncm for gold screws. Trimpou et al. [15] did not report the screw material used. Notably, none of these four studies observed implant fractures. Martin et al. [19] demonstrated that gold screws achieve higher preload values compared to titanium screws under identical torque conditions. Inadequate preload may lead to screw loosening and increased micro-movements at the implant-abutment interface, potentially contributing to unfavorable stress concentration in the coronal region of the implant [20, 21]. This hypothesis is corroborated by Zhang et al. [22], who investigated the CERALOG/PEKK implant-abutment combination in vitro and reported fracture in the coronal third after 4.7 million loading cycles, closely matching the in vivo failure pattern observed in the present study. Following cyclic loading, surviving implants exhibited significantly reduced fracture loads (751 ± 231 N vs. 995 ± 161 N control, *p* = 0.046). Ahmed et al. [23] recently compared PEKK and zirconia abutments for screw-retained crowns on two-piece zirconia implants in vitro and reported that PEKK abutments demonstrated adequate survival for posterior indications, while neither abutment type was recommended for anterior sites. Kohal et al. [24] investigated two-piece zirconia implants with titanium versus zirconia abutment screws and found no significant differences in fracture resistance between groups, suggesting that screw material may have a more relevant effect on preload dynamics than on static fracture resistance. The absence of fractures in the Sterzenbach et al. trial using gold screws [13], despite similar implant geometry and prosthetic design, suggests that screw material may play a relevant role in the biomechanical behavior of the implant-abutment complex. However, the preload dynamics of different screw materials in ceramic implant systems have not been extensively investigated, and this association requires further research.

When compared with other two-piece zirconia implant systems, the survival rate observed in our cohort is notably lower. A recent systematic review by Bazal-Bonelli et al. [2] reported a pooled survival rate of 96.31% across 298 two-piece zirconia implants. Balmer et al. [25] published a multicenter randomized controlled trial comparing 61 two-piece zirconia implants (Straumann PURE Ceramic Implant; Institut Straumann AG) with 61 titanium implants and reported 100% survival for the zirconia group after one year of loading, with no significant differences in marginal bone loss or peri-implant health parameters. Karapataki et al. [26] provided the longest available follow-up data, demonstrating 100% implant survival and no peri-implantitis in 91 two-piece zirconia implants (Patent; Zircon Medical Management) followed for 5–12 years. Tartsch et al. [27] reported the largest retrospective series to date, evaluating 167 two-piece zirconia implants (NobelPearl; Dentalpoint AG and ZERAMEX XT; Dentalpoint AG) with a survival rate of 98.2% after a mean follow-up of 39 months (up to 88 months). Da Silva et al. [28] reported a 12-month survival rate of 98.5% for 65 two-piece ceramic implants (Neodent Zi; Neodent) restored predominantly with cemented prostheses.

Among the earlier published studies about two-piece zirconia implants, Cionca et al. [29, 30] reported 87% survival after one year and 83% after six years (Zeramex T; CeramTec), Brüll et al. [31] demonstrated 96.5% survival after 3 years for a mixed cohort of one-piece and two-piece custom-made Y-TZP implants (ZV3; Zirkon Vision), Payer et al. [32] reported a 2-year survival rate of 93.3% (Ziterion Vario T; Ziterion GmbH) and Becker et al. [33] reported 95.8% survival after two years (ZV3 implants; Zirkon Vision). However, all these studies employed cemented abutments and cemented prosthetic restorations, differing fundamentally from our screw-retained approach. Notably, Beus et al. [34] reported a survival rate of only 75.8% at five years for custom-made ZV3 two-piece zirconia implants and concluded that this particular system could not be recommended for clinical practice, indicating that poor survival is not unique to this cohort but may reflect system-specific factors. Sala et al. [14], who used a comparable prosthetic workflow with PEKK abutments and gold screws on the same implant system, reported a five-year survival rate of 73% at the implant level, with all losses occurring during the osseointegration phase. After definitive restoration placement, both one- and two-piece implants demonstrated a 100% survival rate in their cohort, a pattern consistent with our 36-month observation. The wide range of survival rates reported across different two-piece zirconia implant systems (60.9% to 100%) underscores the critical role of system-specific factors, including surface treatment, implant-abutment connection design, and prosthetic protocol, in determining clinical outcomes.

Soft tissue parameters of the present cohort improved progressively, with mean probing pocket depths decreasing from 2.7 ± 0.7 mm at 12 months to 1.9 ± 0.8 mm at 36 months. These values compare favorably with the probing depths reported by Trimpou et al. [15] (2.53 ± 0.45 mm at 12 months), Giulini et al. [16] (3.7 ± 0.7 mm at 24 months), and Sala et al. [14] (4.1 ± 0.81 mm at five years). Bleeding on probing at the site level decreased from 26% at 12 months to 18% at 36 months, approaching the baseline value of 17.7% recorded at definitive loading. When evaluated at the implant level, peri-implant mucositis was defined as the presence of bleeding on gentle probing without progressive bone loss, in accordance with the 2017 World Workshop consensus [12]. An implant was diagnosed with mucositis if bleeding on probing was recorded at one or more of the six probing sites. Using this criterion, peri-implant mucositis was diagnosed in 7 of 13 clinically examined implants (53.8%) at 36 months, decreasing from 11 of 16 implants (68.8%) at the time of definitive loading. Trimpou et al. [15] reported a comparable BOP of 16.67% at the site level at 12 months, with mucositis present in 68.75% of implants. Giulini et al. [16] observed a BOP rate of 42% at the site level and 84% at the implant level. No sites showed suppuration at any follow-up visit, and no peri-implantitis was diagnosed throughout the observation period, which is consistent with the findings of Trimpou et al. [15], Sala et al. [14], and Karapataki et al. [26], who reported no peri-implantitis in any of their zirconia implant cohorts over follow-up periods of up to 12 years. The mucositis prevalence of 53.8% in our cohort at 36 months is nearly identical to the 53% reported by Sala et al. at five years. Although in vitro data suggest that bacterial plaque accumulates less readily on zirconia than on titanium surfaces [35], the high mucositis rates observed across studies indicate that zirconia implants are not inherently protected from peri-implant soft tissue inflammation. Sala et al. attributed the high mucositis prevalence in their cohort to poor compliance with maintenance programs and emphasized the importance of regular peri-implant maintenance, particularly in the presence of limited keratinized mucosa [36]. Radiographic assessment revealed stable marginal bone levels, with mean DIB values of 1.5 ± 0.9 mm at 36 months. This is comparable to the marginal bone loss reported by Sala et al. (1.6 ± 0.9 mm at five years) and Giulini et al. (1.2 ± 0.9 mm at 24 months), and within the range observed by Balmer et al. [25] for two-piece zirconia implants in their multicenter RCT. Patient satisfaction remained consistently high throughout the observation period. Taken together, these observations suggest that poor oral hygiene was associated with peri-implant mucosal inflammation in individual patients but did not consistently correlate with progressive radiographic bone loss or with implant survival in our cohort. The absence of an association between oral hygiene status and early implant failure supports the interpretation that the observed early failure pattern is related to system-specific rather than patient-specific factors.

It should be noted that the CERALOG Hexalobe system investigated in the present study and in the published clinical literature has been commercially superseded by the CERALOG Progressive-Line (CeramTec Schweiz GmbH, Spreitenbach, Switzerland), which became available in February 2025. In contrast to the CIM-manufactured Hexalobe implants, the Progressive-Line implants are fabricated from hot isostatic pressing (HIP) processed alumina-toughened zirconia (ATZ) blanks by milling and feature a blasted, etched, hydrophilic surface described by the manufacturer as comparable to the CAMLOG Promote surface. Furthermore, the internal connection design has been changed from Hexalobe to Bolt-in-Tube, and a carbon-fiber-reinforced PEEK (VICARBO) abutment screw replaces the previously used titanium or gold screws [37]. These modifications may address the potential contributing factors to implant failure identified across the five available CERALOG studies: surface characteristics, implant material strength, and abutment screw preload dynamics. However, no clinical data on the Progressive-Line are available to date.

Several limitations should be considered when interpreting these results. The relatively small sample size of 24 patients limits statistical power and precludes meaningful inferential subgroup analyses for potential influencing factors such as smoking status, implant position, and bone quality. For the same reason, peri-implant tissue parameters and patient-reported outcomes are presented descriptively across the observation period rather than tested with formal longitudinal statistics; subgroup distributions and longitudinal trends are therefore reported in a hypothesis-generating manner. This study investigated a specific CIM-manufactured two-piece zirconia implant system with screw-retained prosthetic restorations on PEKK abutments, which may restrict generalizability to other zirconia implant systems or prosthetic concepts. The absence of a control group with titanium implants prevents direct comparison with the current standard of care. Furthermore, patients who confirmed implant survival via telephone rather than attending clinical examination represent a potential source of bias, as telephone-based confirmation cannot detect subclinical mechanical or biological complications. As a single-center pilot study, validation through larger multicenter studies is warranted. Extended follow-up through 60 months post-loading is ongoing and will provide further insight into the long-term performance of this implant system. The consistent failure pattern observed across independent study groups may suggest a system-related factor rather than operator-dependent variables.

## Conclusion

In conclusion, the CERALOG Hexalobe two-piece CIM-manufactured zirconia implant system with screw-retained restorations on PEKK abutments demonstrated an unsatisfactory overall survival rate of 60.9%, with all failures concentrated before or shortly after definitive loading. This failure pattern is consistent across the available clinical studies using this implant system. No further implant losses were recorded between 12 and 36 months, and implants that achieved successful osseointegration demonstrated stable marginal bone levels through 36 months. However, persistent peri-implant mucositis in approximately half of the surviving implants at 36 months underlines the need for peri-implant maintenance. The early post-loading period therefore represents the critical determinant of long-term implant survival in this system, and the roles of CIM surface characteristics and abutment screw material as potential contributing factors to early failures warrant further investigation. Extended follow-up through 60 months is ongoing.

## Data Availability

The datasets used and analyzed during the current study are available from the corresponding author on reasonable request.

## Abbreviations

ATZ: Alumina-toughened zirconia
BOP: Bleeding on probing
CIM: Ceramic injection molding
DIB: Distance from implant shoulder to first bone contact
DRKS: Deutsches Register Klinischer Studien
MGI: Modified gingival index
PEEK: Polyetheretherketone
PEKK: Polyetherketoneketone
PPD: Probing pocket depth
PROMs: Patient-reported outcome measures
RCT: Randomized controlled trial
Y-TZP: Yttrium-stabilized tetragonal polycrystalline zirconia
